# Author Correction: Macrophage-dependent IL-1β production induces cardiac arrhythmias in diabetic mice

**DOI:** 10.1038/s41467-021-27508-w

**Published:** 2021-12-08

**Authors:** Gustavo Monnerat, Micaela L. Alarcón, Luiz R. Vasconcellos, Camila Hochman-Mendez, Guilherme Brasil, Rosana A. Bassani, Oscar Casis, Daniela Malan, Leonardo H. Travassos, Marisa Sepúlveda, Juan Ignacio Burgos, Martin Vila-Petroff, Fabiano F. Dutra, Marcelo T. Bozza, Claudia N. Paiva, Adriana Bastos Carvalho, Adriana Bonomo, Bernd K. Fleischmann, Antonio Carlos Campos de Carvalho, Emiliano Medei

**Affiliations:** 1 Institute of Biophysics Carlos Chagas Filho, Universidade Federal do Rio de Janeiro, Rio de Janeiro, 21941-902 Brazil; 2 LIRS-Laboratory of Immunoreceptors and Signaling, Universidade Federal do Rio de Janeiro, Rio de Janeiro, 21941-902 Brazil; 3 Instituto de Microbiologia, Universidade Federal do Rio de Janeiro, Rio de Janeiro, 21941-902 Brazil; 4 Center for Biomedical Engineering, University of Campinas, Campinas, 13.083-970 Brazil; 5 Departamento de Fisiologı ´a, Facultad de Farmacia, Universidad del País Vasco UPV/EHU, 01006 Vitoria, Spain; 6 Institute of Physiology I, Life and Brain Center, University of Bonn, Bonn, D-53127 Germany; 7 Centro de Investigaciones Cardiovasculares, Conicet La Plata, Facultad de Ciencias Médicas, Universidad Nacional de La Plata, La Plata, 1900 Argentina; 8 FIOCANCER/ VPPLR/FIOCRUZ, FIOCRUZ-Manguinhos, Rio de Janeiro, 21040-360 Brazil; 9 National Center for Structural Biology and Bioimaging—CENABIO/UFRJ, Rio de Janeiro, 21941-902 Brazil

Correction to: *Nature Communications* 10.1038/ncomms13344, published online 24 November 2016.

In the original version of this article the “Method” section stated an incorrect provenance for the cardiomyocytes Cor.4U. These were not derived from human iPS cells, as stated in the original article, but were derived from the human embryonic stem (hES) cell line RUES2, as has recently been determined following short tandem repeat testing carried out by Ncardia AG (formerly Axiogenesis). This does not alter the findings and interpretation of the data, as the cells are bone fide human pluripotent cell derived cardiomyocytes.

The original sentence in the “Methods” section read: “MEA measurements in cardiomyocytes derived from induced pluripotent stem cells (iPS) (Cor.4U—Axiogenesis, Germany) were performed”. The sentence now reads: “MEA measurements in cardiomyocytes derived from the hES cell line RUES2 (Cor.4U—Axiogenesis (now Ncardia AG, Germany)) were performed”.

The legend of Fig. 2k originally read: ‘Representative traces of field potential in human iPS-derived cardiomyocytes highlighting effect of IL-1β on the field potential duration (FPD)’. This now reads: ‘Representative traces of field potential in hES cell-derived cardiomyocytes highlighting effect of IL-1β on the field potential duration (FPD)’.

The legend of Fig. 3e originally read: ‘Representative traces of field potential in human iPS-derived cardiomyocytes highlighting inhibitory influence of CamKII with AIP of the IL-1β effect on the field potential duration (FPD)’. It now reads: ‘Representative traces of field potential in hES cell-derived cardiomyocytes highlighting inhibitory influence of CamKII with AIP of the IL-1β effect on the field potential duration (FPD)’.

In the “Results” section the sentence that read “We, therefore, tested IL-1β effects in human induced pluripotent stem-cell derived cardiomyocytes (hIPS-CM) using field potential duration (FPD) measurement. Our experiments revealed longer FPD on IL-1β treated hIPS cell-CM, when compared with untreated hIPS-CM” now reads “We, therefore, tested IL-1β effects in human embryonic stem-cell derived cardiomyocytes (hES-CM) using field potential duration (FPD) measurement. Our experiments revealed longer FPD on IL-1β treated hES-CM, when compared with untreated hES-CM”.

In the “Results” section the sentence that read “Additionally, in order to elucidate whether the CaMKII inhibition also could have a key role in human cells, hIPS-CM were exposed to IL-1β in the absence or in the presence of a selective CaMKII inhibitor (autocamtide—2—related inhibitory peptide (AIP)). In this setting, it was observed that the CaMKII inhibition was able to prevent the IL-1β-induced longer FPD in hIPS-CM (Fig. 3e, f)”. now reads: “Additionally, in order to elucidate whether the CaMKII inhibition also could have a key role in human cells, hES-CM were exposed to IL-1β in the absence or in the presence of a selective CaMKII inhibitor (autocamtide-2-related inhibitory peptide (AIP)). In this setting, it was observed that the CaMKII inhibition was able to prevent the IL-1β-induced longer FPD in hES-CM (Fig. 3e, f)”.

In the Acknowledgements, the sentence that read: “We thank Axiogenesis AG for providing hiPS-derived cardiomyocytes and Genentech, which authorized the use Nlrp3−/− mice” now reads: “We thank Axiogenesis AG (now Ncardia AG) for providing hES cell-derived cardiomyocytes and Genentech, which authorized the use Nlrp3−/− mice”. These errors have now been corrected in the HTML and PDF version of the article.

