# Supplementary material for: Macrophage-dependent IL-1β production induces cardiac arrhythmias in diabetic mice
Source: Nat Commun. 2016 Nov 24;7:13344. doi: 10.1038/ncomms13344 (PMC5123037; doi:10.1038/ncomms13344)
Supplement: Supplementary Information — Supplementary Figures 1-11 and Supplementary Tables 1-3. [file ncomms13344-s1.pdf]

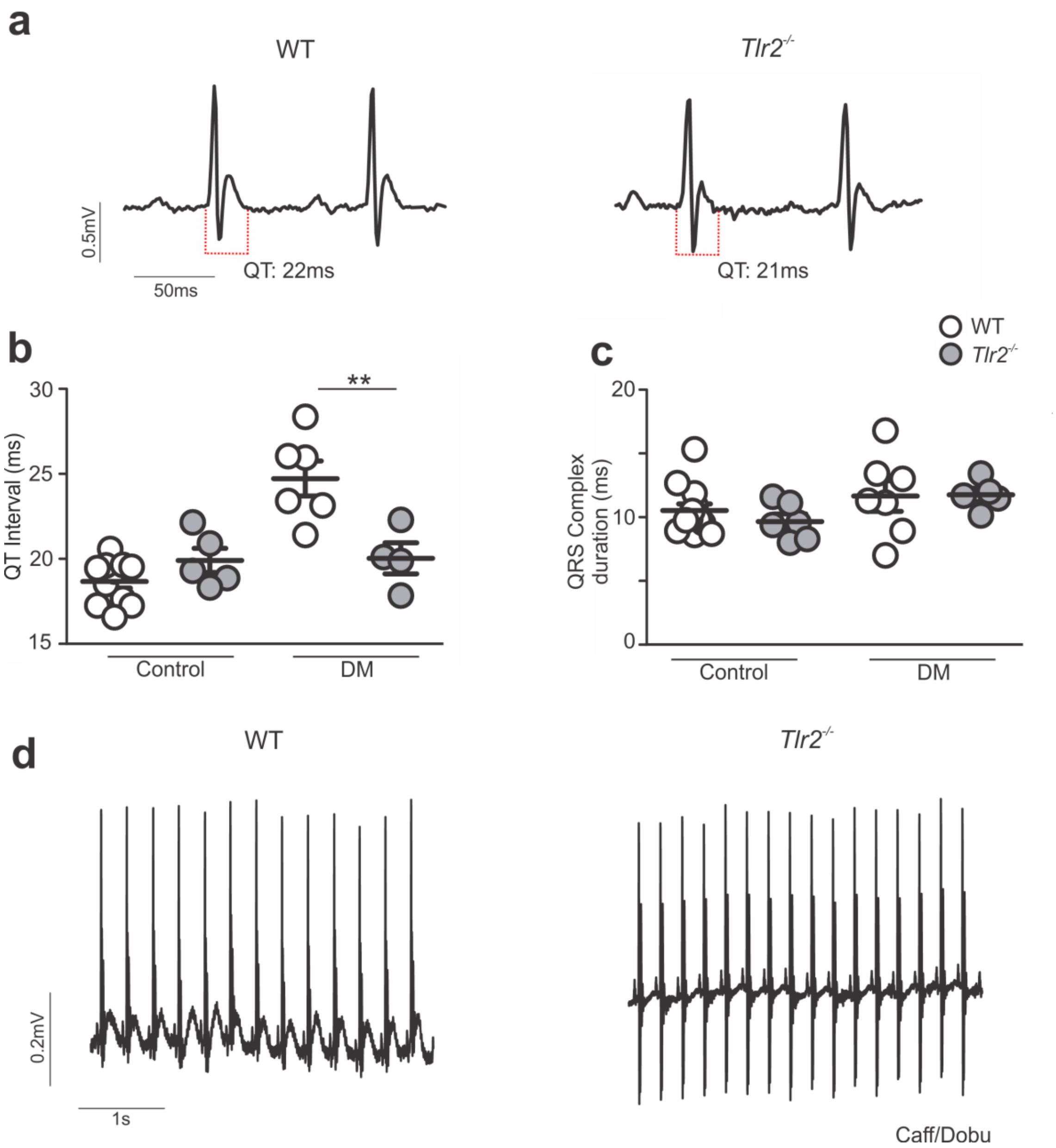

Supplementary Figure 1 Electrocardiogram (ECG) parameters from WT and TLR2 mice.

(**a**) Representative ECG traces of non-diabetic WT and T*lr2*$^{-/-}$ mice 60 days after vehicle. (**b,c**) Summary of main ECG parameters: QRS and QT interval duration. (**d**) Representative traces of arrhythmic vulnerability test induced by caffeine and dobutamine (Caff/Dobu) showing a normal ECG in non-diabetic WT and TLR2 mice (n=WT: 10; *Tlr2*$^{-/-}$: 5; WT+DM: 6; *Tlr2*$^{-/-}$+DM: 4). Scatter plot shows values from individual mice, horizontal bars are the means and error bars represents SEM. ** represent $P < 0.01$ versus WT+DM (unpaired t-test).

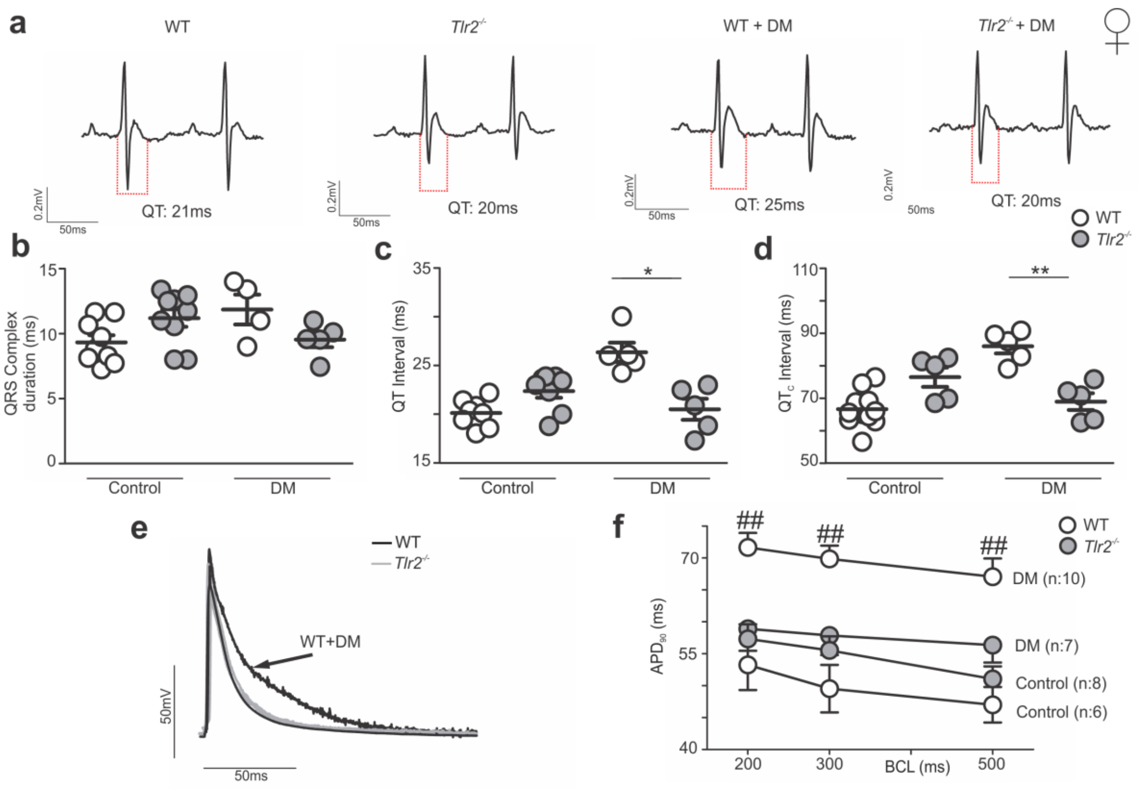

Supplementary Figure 2 TLR2 regulates cardiac electrical alterations induced by diabetes in female mice, showing no gender specificity

(**a**) Representative ECG traces of experimental groups after 60 days of streptozotocin or vehicle. (**b-d**) Summary of main ECG parameters: QRS duration; QT interval duration and corrected QT interval (QTc) (n=WT: 8; *Tlr2*$^{-/-}$: 9; WT+DM: 5; *Tlr2*$^{-/-}$+DM: 5). (**e**) Representative action potential traces from left endocardium at 300 ms basic cycle length (BCL) stimulation. (**f**) The graph summarizes the action potential duration at 90 percent of repolarization (APD_{90}) at different BCL. Scatter plot shows values from individual mice, horizontal bars are the means and error bars represents SEM. * and ** represent, respectively, $P < 0.05$ and $P < 0.01$, versus WT+DM (unpaired t-test). ## represent $P < 0.01$ versus WT+DM (Bonferroni's *post* test following two-way ANOVA with).

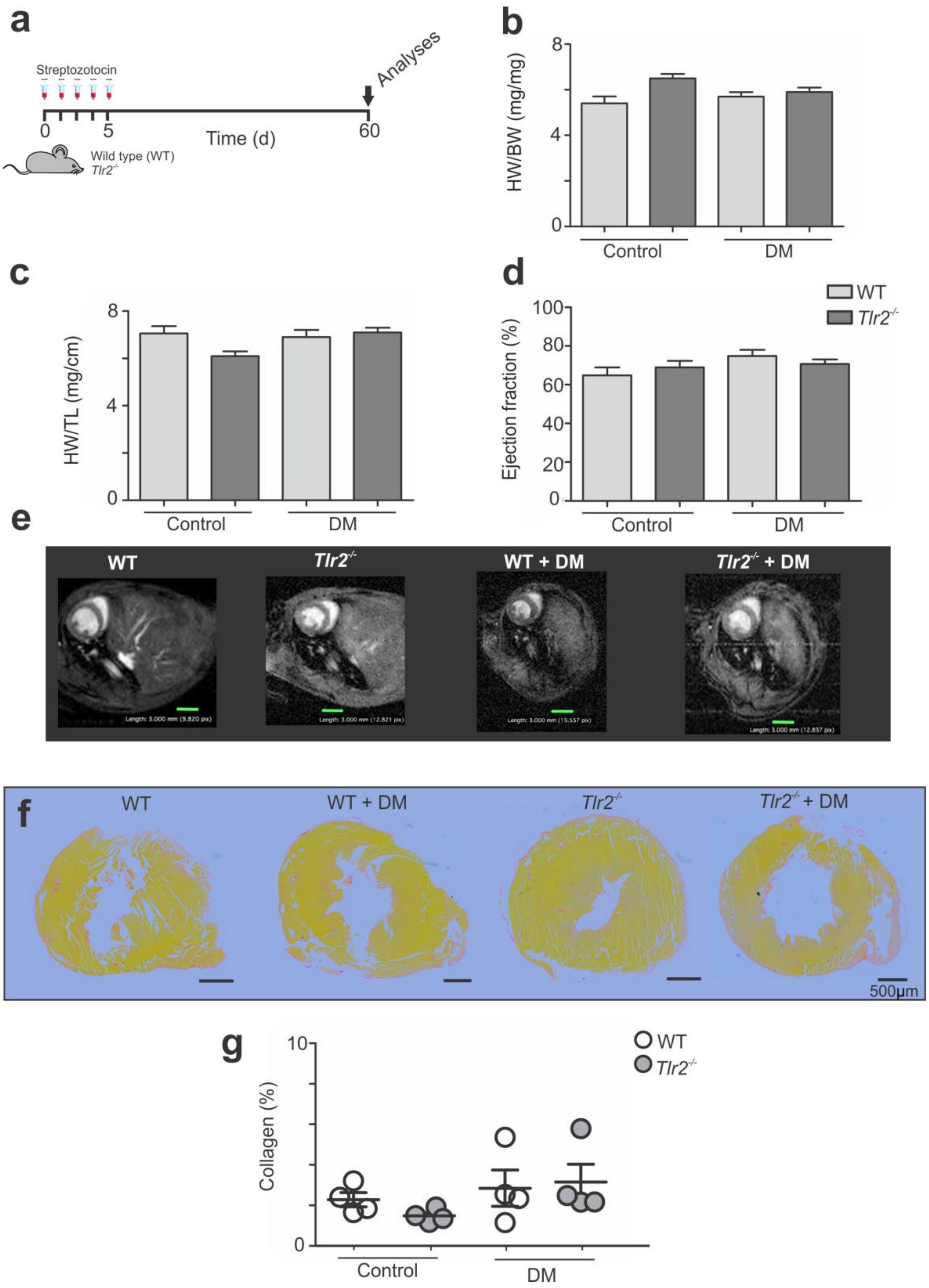

Supplementary Figure 3 Diabetes and TLR2 do not change heart properties

(**a**) Experimental protocol: diabetes (DM) was induced in wild type (WT) and toll-like receptor 2 *knock-out* mice (*Tlr2*$^{-/-}$) by 5 daily i.p. injections of streptozotocin (STZ) (50 mg/kg) and the parameters were analyzed 60 days after the protocol started. (**b,c**) Graphs summarize heart weight (HW) / body weight (BW) ratio and HW / tibia length

(TL) ratio from at least 10 mice from each experimental group. (**d**) Graph summarizes ejection fraction values from at least 4 mice from each experimental group obtained by functional 7T magnetic resonance imaging (MRI). (**e**) Representative MRI images (n=5 per group). Scale bar: 3000 mm. (**f**) Representative images of transverse sections of the heart stained with picrosirius red for collagen after 60 days of streptozotocin or vehicle. Scale bar: 500 μm. (**g**) Graph summarizes the percentage of collagen in the heart. The results are expressed as mean ± SEM (n=4 per group).

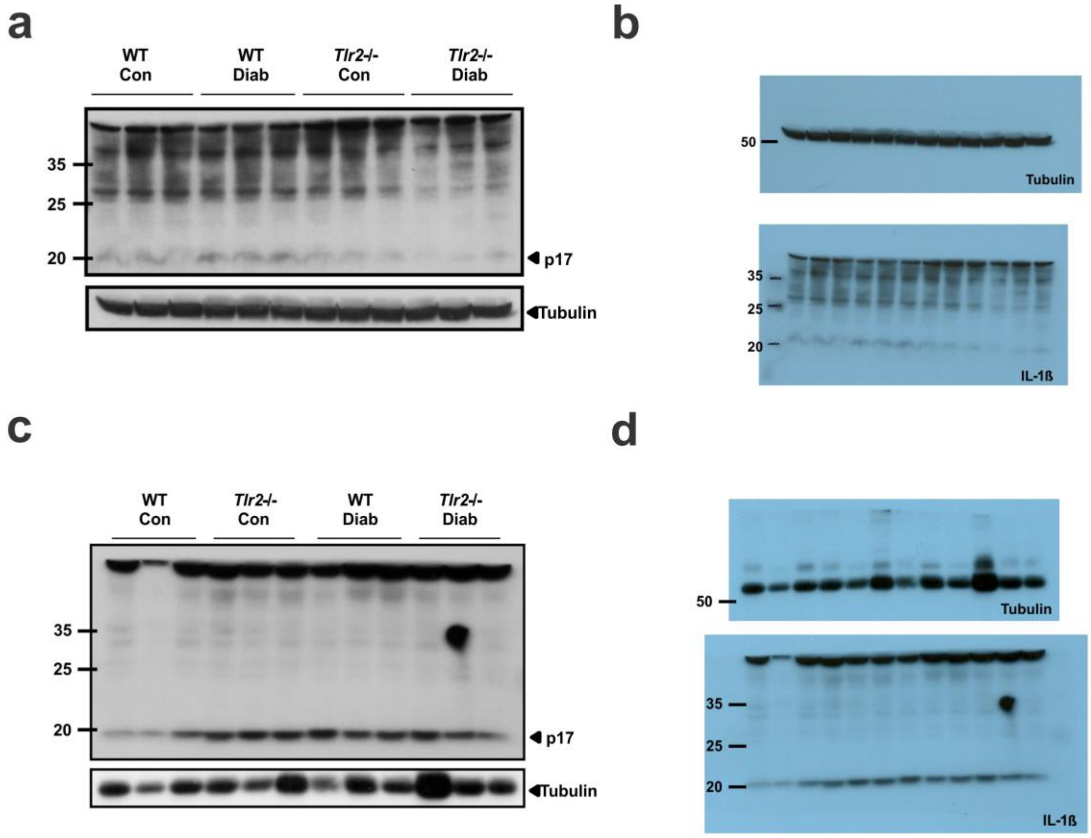

Supplementary Figure 4 Original Images of Uncropped Western blots.

(**a-d**) Uncropped blots appearing in Figures 1**i,j**.

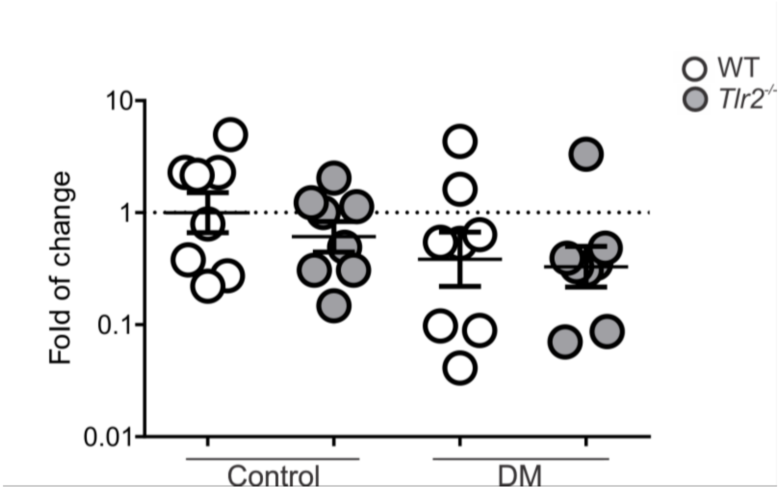

Supplementary Figure 5 IL-1β gene expressions.

Cardiac IL-1β mRNA expression in experimental groups (n=control: 8; *Tlr2*$^{-/-}$, 8; WT DM: 7; *Tlr2*$^{-/-}$ DM: 8). The scatter plot shows values from individual heart. Horizontal bars are the means and error bars represent SEM.

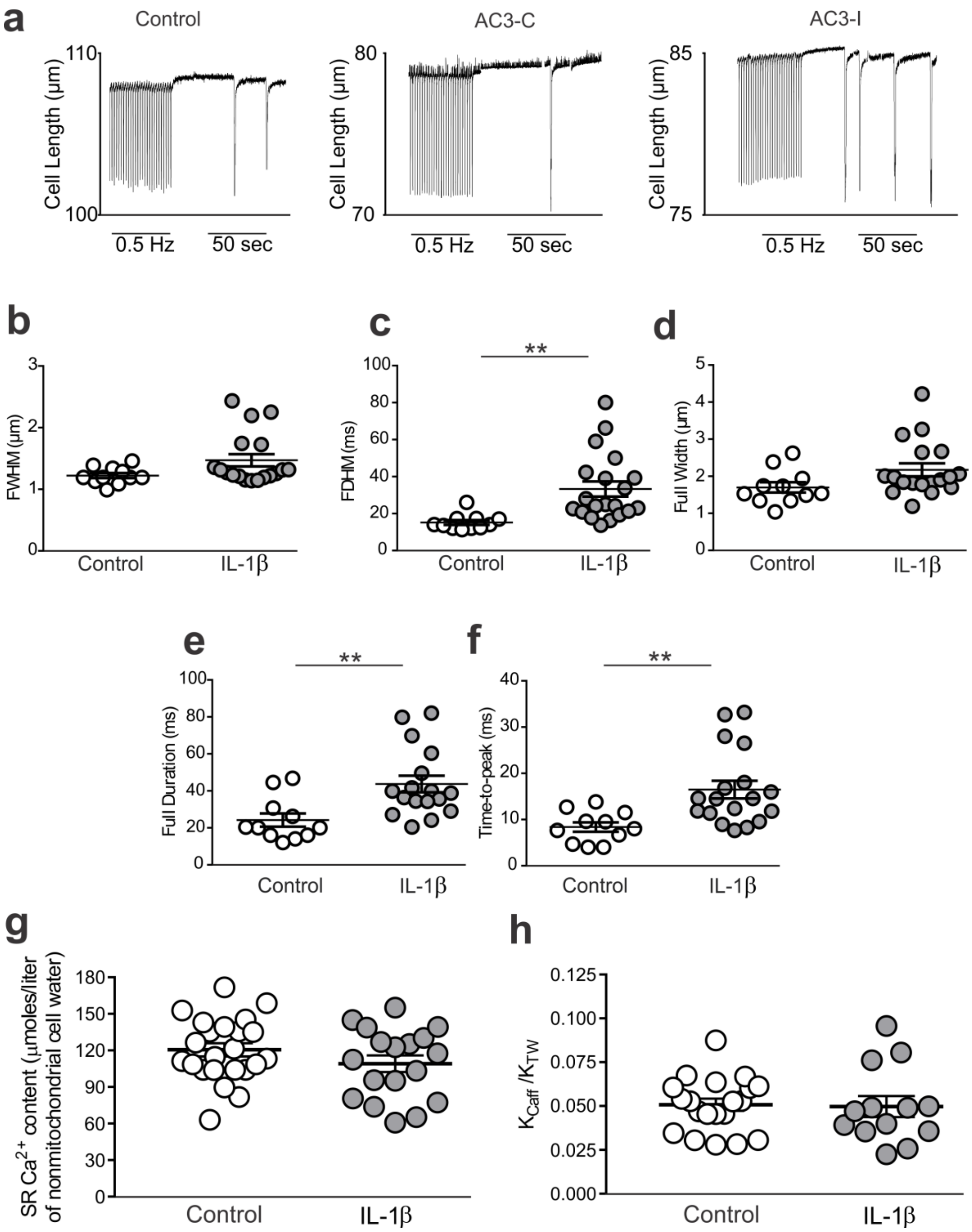

Supplementary Figure 6 Interleukin-1β (IL-1β) effects on isolated murine cardiomyocytes

(**a**) Representative traces of cell shortening in isolated mice cardiomyocyte after 24 h of dissociation, in which spontaneous contractions developed after pacing (0.5 Hz) interruption, can be observed in control situations. (**b-f**) Analyses of calcium sparks in cardiomyocytes after 24 h incubation in the absence or presence of IL-1β: (**b**) Full width at half-maximum amplitude (FWHM) (**c**) Full duration at half-maximum amplitude (FDHM). (**d**) Full width. (**e**) Full duration. (**f**) Time to peak. (**g**) Sarcoplasmatic

reticulum calcium content (**h**) Ratio of the rate constant of [Ca^{2+}] decline during transients evoked by 10 mM of caffeine (K-Cf) and electrical stimulation (K-TW). The scatter plot shows values from individual cells from 4 hearts (n=11-18 cells/ group). Horizontal bars are the means and error bars represents SEM. *; ** and *** represent, respectively, $P < 0.05$; $P < 0.01$ and $P < 0.001$ (unpaired t-test). # and ## represent, respectively, $P < 0.05$ and $P < 0.01$, (Bonferroni's *post* test following one-way ANOVA with). (**e**) spark fluorescence amplitude (F/F_0).

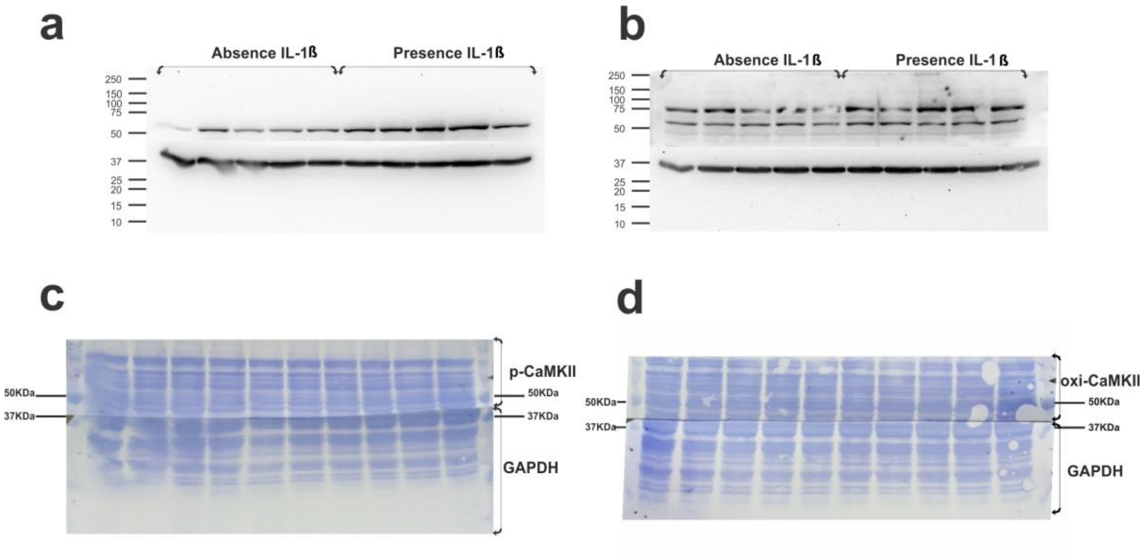

Supplementary Figure 7 Original Images of Uncropped Western blots

(**a-d**) Uncropped blots appearing in Figures 3**a,b**

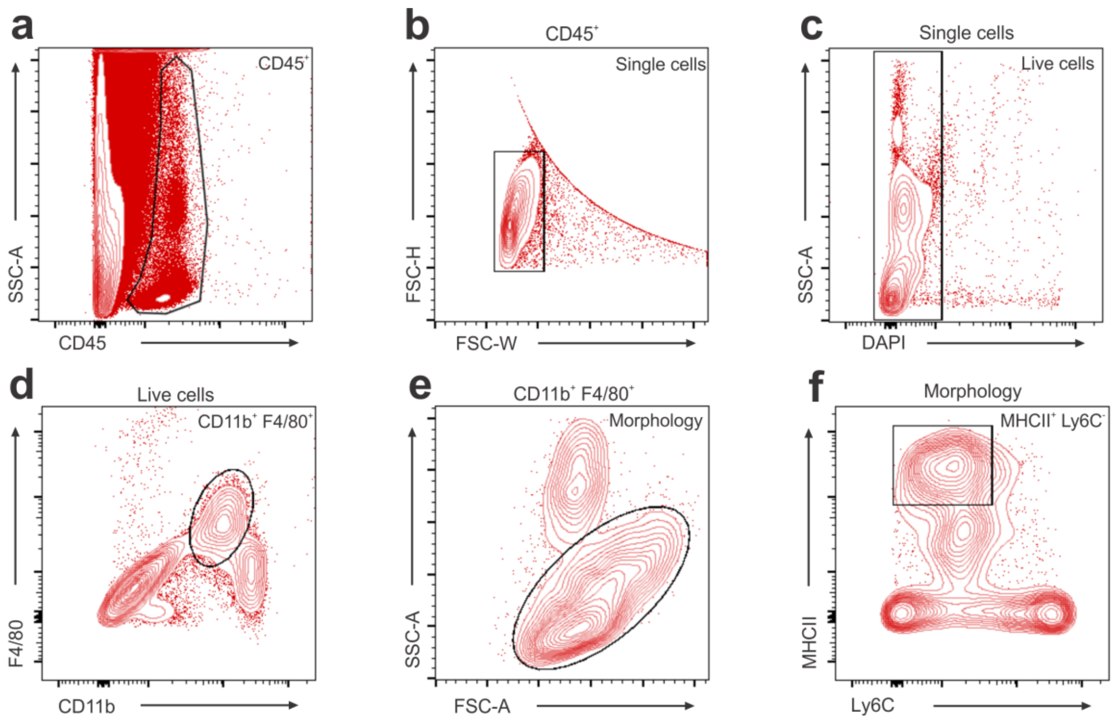

Supplementary Figure 8 Flow cytometry gating strategy for identifying cardiac macrophages

Cardiac tissue suspensions containing single cells were prepared and labeled. (**a**) CD45+ leukocytes were identified. (**b**) Doublets were excluded using FSC-H and FSC-W. (**c**) Using DAPI and granularity (SSC-A), dead cells were excluded. (**d**) Live cells were gated with F4/80+ and CD11b+, further (**e**) cell size (FSC-A) and granularity (SSC-A) were used for cellular morphology selection, and then macrophages were identified (**f**) with MHC-II and Ly6c expression.

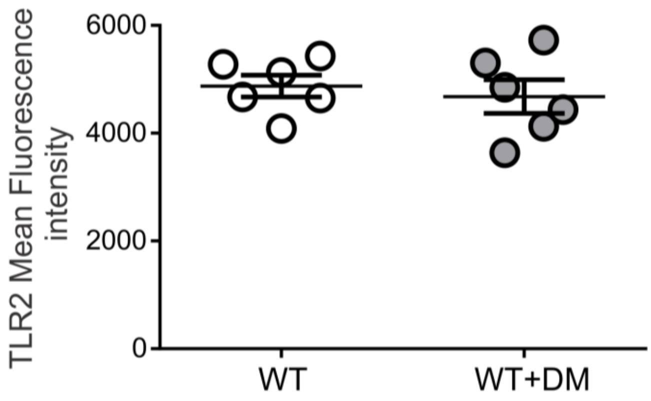

Supplementary Figure 9 TRL2 Mean Fluorescence Intensity in cardiac macrophages.

TLR2 fluorescence intensity analyses in cardiac macrophages sorted from cardiac tissues suspensions (n=6 per group). The scatter plot shows values from individual heart. Horizontal bars are the means and error bars represent SEM.

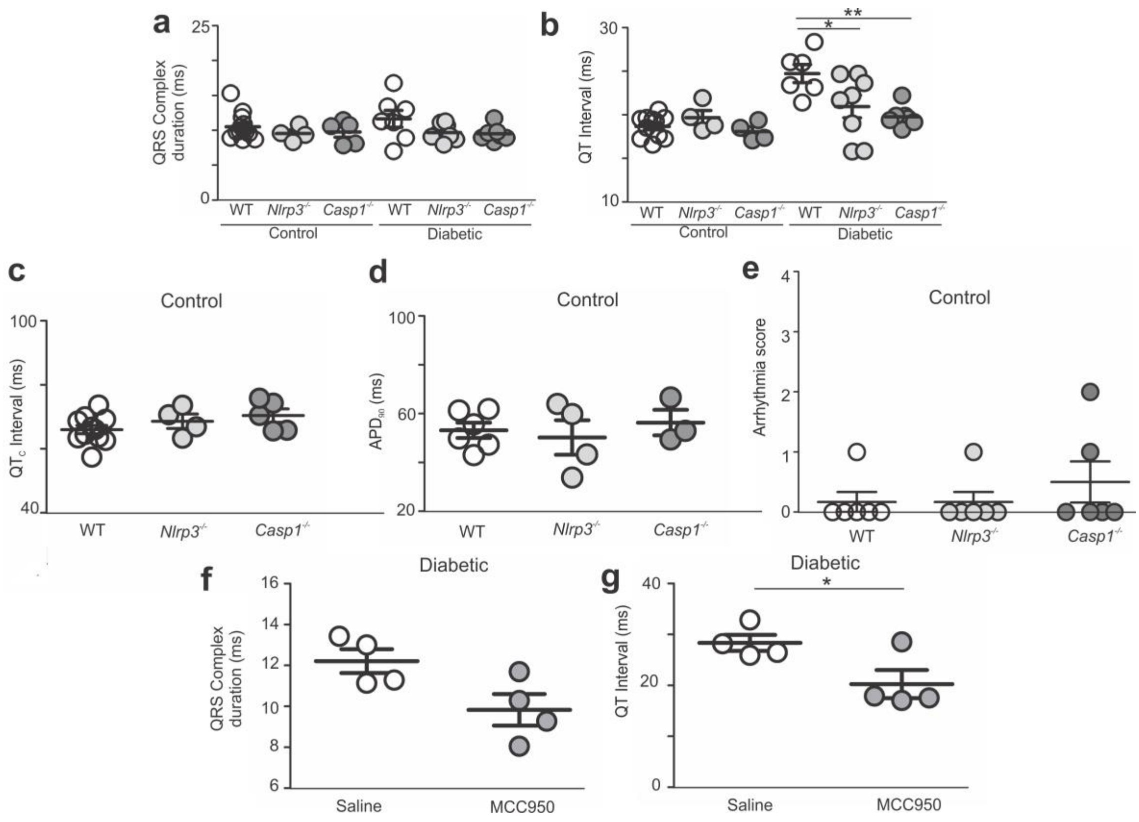

Supplementary Figure 10 ECG parameters, action potential duration and arrhythmia vulnerability test in WT, *Nlrp3*$^{-/-}$, *Casp1*$^{-/-}$ and MCC-950 treated mice

(**a,b**) Summary of main ECG parameters: QRS and QT interval duration (n=WT: 9; *Nlrp3*$^{-/-}$: 4; *Casp1*$^{-/-}$, 5; WT DM: 7; *Nlrp3*$^{-/-}$ DM: 8; *Casp1*$^{-/-}$ DM: 7). (**c,d**) The corrected QT interval and action potential duration at 90 percent of repolarization (APD_{90}) at 300 ms BCL from non-diabetic groups (referred to **Fig. 6** of the main manuscript) (n=WT: 5; *Nlrp3*$^{-/-}$: 4; *Casp1*$^{-/-}$, 3). (**e**) Arrhythmia scores win the Caff/Dobu test in non-diabetic WT, *Nlrp3*$^{-/-}$ and *Casp1*$^{-/-}$ mice (n=WT: 6; *Nlrp3*$^{-/-}$: 6; *Casp1*$^{-/-}$, 6). **(f,g)** Summary of QRS and QT interval duration in diabetic mice treated for 14 days with saline or MCC-950 (n=4/ per group). Scatter plot shows values from individual mice, horizontal bars are the means and error bars represents, respectively, SEM. * and ** represent $P < 0.05$ and $P < 0.01$ (unpaired t-test).

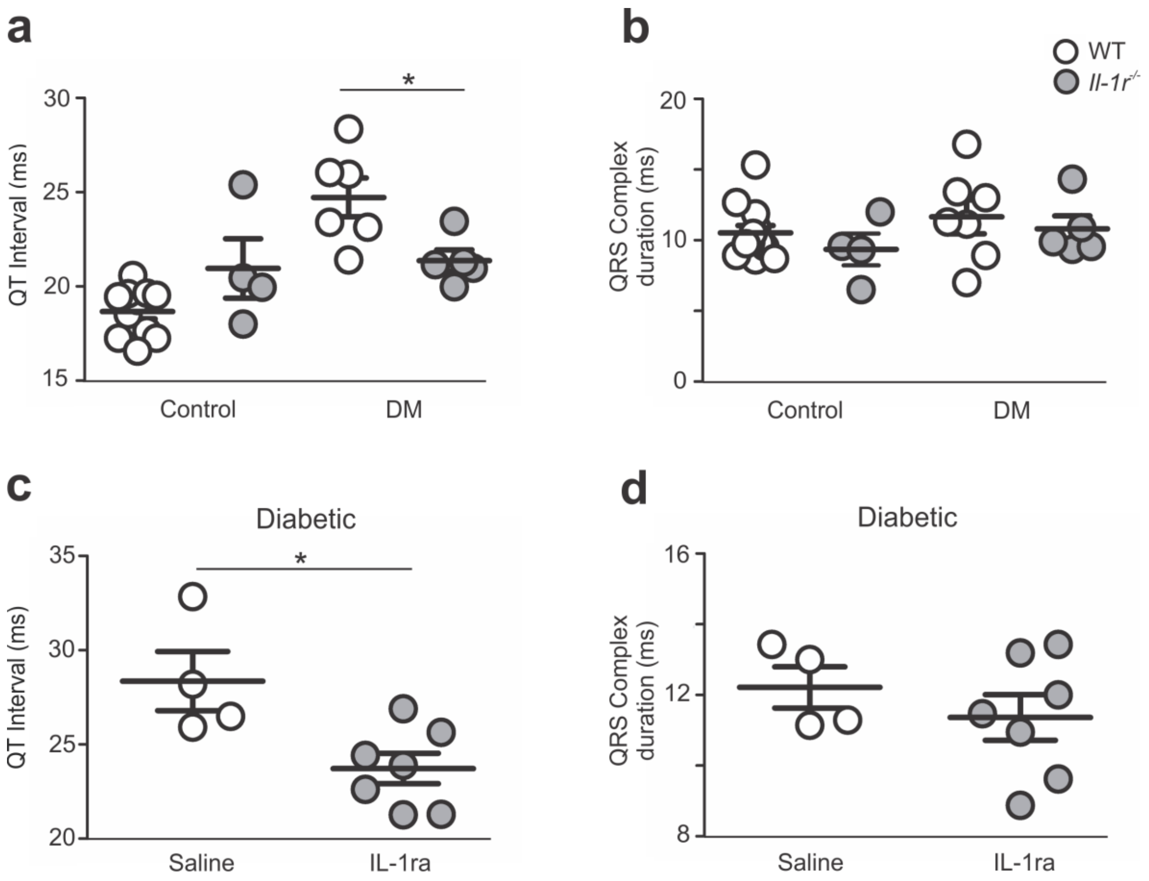

Supplementary Figure 11 ECG parameters and arrhythmia vulnerability test in *IL-1r*$^{-/-}$ mice and in WT mice treated with IL-1ra

(**a,b**) QT interval and QRS duration in WT and *IL-1r*$^{-/-}$ mice (n=WT: 9; *IL-1r*$^{-/-}$: 4; WT DM: 7; *IL-1r*$^{-/-}$ DM: 5) (**c,d**) QT interval and QRS duration in WT diabetic mice treated for 14 days with saline or IL-1ra (n=Saline: 4; IL-1ra: 7). Scatter plot shows values from individual mice, horizontal bars are the means and error bars represent SEM. * represent $P < 0.05$ (unpaired t-test).

Supplementary Table 1. Blood Glucose and Insulin levels

	WT	WT+DM	*P* value	*TRL2*$^{-/-}$	*TLR2*$^{-/-}$ +DM	*P* value
Glucose (mg/dL)	102.6 ±5.2	343.3 ± 37.7	0.0006	95.29 ± 2.9	471.0 ± 26.9	< 0.0001
Insulin (µlU/mL)	12.7 ± 0.7	10.1 ± 0.9	0.0471	11.0 ± 0.5	9.5 ± 0.4	0.0741

n: WT: 7; WT+DM: 8; *Tlr*2$^{-/-}$: 6; *Tlr*2$^{-/-}$+DM: 6.

Supplementary Table 2. Blood Glucose and Insulin levels and Cardiac Biometry

	Nlrp3$^{-/-}$	*Nlrp3*$^{-/-}$ +DM	*P* value	*Casp1*$^{-/-}$	*Casp1*$^{-/-}$ +DM	*P* value
Glucose (mg/dL)	132.6 ± 9.4	361.0 ± 20.3	< 0.0001	122.8 ± 6.0	337.4 ± 30.7	0.0006
Insulin (μlU/mL)	12.7 ± 1.4	10.2 ± 0.8	0.1761	16.7 ± 2.5	10.9 ± 1.0	0.0542
HW/BW (mg/g)	6.4 ± 0.4	5.9 ± 0.2	0.3894	6.5 ± 0.6	6.4 ± 0.3	0.7787
HW/TL (mg/cm)	8.2 ± 0.6	7.2 ± 0.3	0.1449	9.1 ± 0.7	8.4 ± 0.6	0.4538

For glucose and Insulin: **n**: *Nlrp3*$^{-/-}$: 7;*Nlrp3*$^{-/-}$+DM: 6; *Casp*1$^{-/-}$: 7; *Casp*1$^{-/-}$+DM: 7. For Cardiac Biometry: **n**: *Nlrp3*$^{-/-}$: 9;*Nlrp3*$^{-/-}$+DM: 11; *Casp*1$^{-/-}$: 9; *Casp*1$^{-/-}$+DM: 13.

Supplementary Table 3. Blood Glucose and Insulin levels and Cardiac Biometry

	IL-1r$^{-/-}$	*IL-1r*$^{-/-}$+DM	*P* value
Glucose (mg/dL)	143.3 ± 13.1	387.7 ± 25.3	< 0.0001
Insulin (µlU/mL)	12.9 ± 1.3	9.0 ± 0.9	0.0403
HW/BW (mg/g)	5.4 ± 0.2	5.2 ± 0.3	0.4400
HW/TL (mg/cm)	6.5 ± 0.2	7.1 ± 0.4	0.2872

For glucose and Insulin: **n**: *lL-1r*$^{-/-}$: 6; *IL-1r*$^{-/-}$+DM: 6. For Cardiac Biometry: **n**: *lL-1r*$^{-/-}$: 5; *IL-1r*$^{-/-}$+DM: 5.
